# Heterologous ChAdOx1 nCoV-19 and BNT162b2 prime-boost vaccination elicits potent neutralizing antibody responses and T cell reactivity against prevalent SARS-CoV-2 variants

**DOI:** 10.1016/j.ebiom.2021.103761

**Published:** 2021-12-17

**Authors:** Rüdiger Groß, Michelle Zanoni, Alina Seidel, Carina Conzelmann, Andrea Gilg, Daniela Krnavek, Sümeyye Erdemci-Evin, Benjamin Mayer, Markus Hoffmann, Stefan Pöhlmann, Weimin Liu, Beatrice H. Hahn, Alexandra Beil, Joris Kroschel, Bernd Jahrsdörfer, Hubert Schrezenmeier, Frank Kirchhoff, Jan Münch, Janis A. Müller

**Affiliations:** a Institute of Molecular Virology, Ulm University Medical Center, 89081, Ulm, Germany; b Institute for Epidemiology and Medical Biometry, Ulm University, Ulm, Germany; c Infection Biology Unit, German Primate Center – Leibniz Institute for Primate Research, Göttingen, Germany; d Faculty of Biology and Psychology, Georg-August-University Göttingen, Göttingen, Germany; e Department of Microbiology and Department of Medicine, University of Pennsylvania, Philadelphia, USA; f Central Department for Clinical Chemistry, University Hospital Ulm, 89081, Ulm, Germany; g Institute for Transfusion Medicine, Ulm University, 89081, Ulm, Germany; h Institute for Clinical Transfusion Medicine and Immunogenetics Ulm, German Red Cross Blood Services Baden-Württemberg-Hessen and University Hospital Ulm, 89081, Ulm, Germany; i Core Facility Functional Peptidomics, Ulm University Medical Center, 89081, Ulm, Germany

**Keywords:** COVID-19, Delta, B.1.617.2, immunity, heterologous vaccination

## Abstract

**Background:**

Heterologous COVID-19 vaccination regimens combining vector- and mRNA-based vaccines are already administered, but data on solicited adverse reactions, immunological responses and elicited protection are limited.

**Methods:**

To evaluate the reactogenicity and humoral as well as cellular immune responses towards most prevalent SARS-CoV-2 variants after a heterologous ChAdOx1 nCoV-19 BNT162b2 prime-boost vaccination, we analysed a cohort of 26 clinic employees aged 25-46 (median 30.5) years who received a ChAdOx1 nCoV-19 prime followed by a BNT162b2 boost after an 8-week interval. Serological data were compared to a cohort which received homologous BNT162b2 vaccination with a 3-week interval (14 individuals aged 25-65, median 42).

**Findings:**

Self-reported solicited symptoms after ChAdOx1 nCoV-19 prime were in line with previous reports and more severe than after the BNT162b2 boost. Antibody titres increased significantly over time resulting in strong neutralization titres two weeks after the BNT162b2 boost and subsequently slightly decreased over the course of 17 weeks. At the latest time point measured, all analysed sera retained neutralizing activity against the currently dominant Delta (B.1.617.2) variant. Two weeks post boost, neutralizing activity against the Alpha (B.1.1.7) and immune-evading Beta (B.1.351) variant was ∼4-fold higher than in individuals receiving homologous BNT162b2 vaccination. No difference was observed in neutralization of Kappa (B.1.617.1). In addition, heterologous vaccination induced CD4^+^ and CD8^+^ T cells reactive to SARS-CoV-2 spike peptides of all analysed variants; Wuhan-Hu-1, Alpha, Beta, Gamma (P.1), and Delta.

**Interpretation:**

In conclusion, heterologous ChAdOx1 nCoV-19 / BNT162b2 prime-boost vaccination is not associated with serious adverse events and induces potent humoral and cellular immune responses. The Alpha, Beta, Delta, and Kappa variants of spike are potently neutralized by sera from all participants and reactive T cells recognize spike peptides of all tested variants. These results suggest that this heterologous vaccination regimen is at least as immunogenic and protective as homologous vaccinations and also offers protection against current variants of concern.

**Funding:**

This project has received funding from the European Union's Horizon 2020 research and innovation programme, the German Research Foundation, the BMBF, the Robert Koch Institute (RKI), the Baden-Württemberg Stiftung, the county of Lower Saxony, the Ministry for Science, Research and the Arts of Baden-Württemberg, Germany, and the National Institutes of Health.

**Research in context**

**Evidence before this study**

Heterologous vaccination against COVID-19, combining vector- and mRNA-based vaccines, is now part of the applied vaccination regimens in several countries in response to changing recommendations and vaccine shortages. However, evidence on tolerability, immunogenicity or efficacy of such regimens especially against the Delta variant is limited.

**Added value of this study**

Our study of a cohort of 26 clinic employees adds to the accumulating evidence that solicited adverse reactions for most participants receiving an 8-week interval BNT162b2 boost was lower compared to the ChAdOx1 nCoV-19 prime. Importantly, heterologous vaccination resulted in high levels of IgG, IgA, IgG/M, and potent neutralizing antibody titres. Sera obtained after full vaccination neutralized Alpha, Beta and Kappa SARS-CoV-2 spike variants with greater potency than after homologous BNT162b2 vaccination. In addition, neutralizing titres against Delta were detected in all participants 3 months after the boost vaccination. SARS-CoV-2 spike-reactive CD4^+^ and CD8^+^ T cells were regularly elicited by this vaccination regimen, and also responded to Alpha, Beta, Gamma and Delta variants. In conclusion, we provide evidence that in young adults, vaccination with ChAdOx1 nCoV-19 and BNT162b2 in a heterologous prime-boost regimen is well-tolerated and induces potent Delta neutralizing antibody responses still detectable after 3 months as well as T cell reactivity also against variants.

**Implications of all the available evidence**

Combined with published evidence on tolerability and immunogenicity of heterologous vaccination against COVID-19, combining vector- and mRNA-based vaccines is a promising alternative to homologous regimens. The high levels of neutralizing titres and potent T cell responses induced by this regimen, especially against the dominant Delta variant, may be highly beneficial in light of emerging variants showing increased immune evasion. These results thus have direct implications for policy makers, physicians and patients who are offered the choice of such a regimen and offer perspectives to boost immunity in future vaccine updates.

Alt-text: Unlabelled box

## Introduction

The first cases of the coronavirus disease 2019 (COVID-19) were reported to the World Health Organization on December 31^st^ 2019,^1^ and within 93 days the causative severe acute respiratory syndrome coronavirus 2 (SARS-CoV-2) had infected over 1 million people worldwide.^2^ Only 250 days later, the first person received a COVID-19 vaccine outside a clinical trial. To date, vaccination is considered the key strategy for ending the pandemic.^3^ Approved vaccines among others include the adenovirus-based ChAdOx1 nCoV-19 (Vaxzevria, AstraZeneca) and mRNA-based BNT162b2 (Comirnaty, BioNTech/Pfizer), which induce humoral and cellular immunological responses^4^, ^5^, ^6^, ^7^ (and preprint:^8^), showed high efficacy in clinical trials,^9^^,^^10^ and potent protection from COVID-19 in real-world settings.^11^^,^^12^ However, the occurrence of rare thrombotic events with thrombocytopenia after ChAdOx1 nCoV-19 vaccinations, especially in individuals younger than 60 years, associated with the generation of auto-platelet factor 4 antibodies, halted vaccination of this group with ChAdOx1 nCoV-19 in some countries.^13^, ^14^, ^15^ As a consequence, several public health agencies recommended boost vaccinations of individuals already primed with ChAdOx1 nCoV-19 to be carried out in a heterologous regimen with an mRNA vaccine.^16^ In some countries, however, such a regimen is still not applied due to limited data on safety and immunogenicity. Moreover, it remains unclear whether a heterologous boosting schedule is associated with similar^17^, ^18^, ^19^ or greater^20^ reactogenicity compared to homologous vaccination. Finally, while robust immune responses are elicited by heterologous vaccination regimens with a 8-12 week interval,^17^, ^18^, ^19^^,^^21^ there is limited knowledge about humoral and cellular protection against the variants of concern (VOCs), especially the most prevalent Delta (B.1.617.2) variant.^19^^,^^21^, ^22^, ^23^ Further, published studies have not investigated how immunity develops several months after vaccination. Here, we examined a cohort of 26 individuals (16 female, 10 male; median age 30.5, range 25-46) who received ChAdOx1 nCoV-19 prime and, due to changing recommendations in Germany,^16^ a BNT162b2 boost vaccination with a 56-60 day interval and evaluated solicited adverse reactions, humoral and cellular immune responses against several spike variants, including that of the dominant Delta VOC up to 3 months after boost vaccination. We further compared serological parameters to a cohort which received homologous BNT162b2 vaccination with a 3-week interval (14 individuals aged 25-65, median 42; Appendix Table 1).

**Table 1:** Study participants.

	Total	m	f	Trans / non-binary
**Participants**	26	10	16	0
**Age median**	30.5 (25-46)	32 (25-46)	30.5 (26-44)	-
**Prior SARS-CoV-2 infection**	1	0	1	0
**Platelet factor 4 autoantibodies (determined in**^32^**)**	0	0	0	0

## Methods

### Study Design

A cohort of 26 individuals aged 25-46 (median 30.5) years who received a ChAdOx1 nCoV-19 prime followed by a BNT162b2 boost after an 8-week interval were analysed for reactogenicity, antibody responses and T cell reactivity. The study size is the result of volunteers which could be enrolled at Ulm University Medical Centre prior to a scheduled prime vaccination date. Initial blood samples were taken up to 2 days before prime vaccination. Participants were eligible for recruitment if they had not been previously vaccinated against SARS-CoV-2 and were scheduled for a ChAdOx1 nCoV-19 prime vaccination. Prior SARS-CoV-2 infection was determined by medical history and measuring anti-SARS-CoV-2-nucleocapsid antibody levels. One convalescent individual was detected and excluded from all statistical analyses (Table 1). Solicited adverse reactions after both prime and boost vaccination were self-reported and ranked by severity and duration. Blood samples were taken from all participants at several follow-up time points throughout the study and analysed for antibodies and T-cell reactivity against SARS-CoV-2 spike. Follow-up consists of collection of blood samples only and is ongoing as part of a long-term study for this cohort, data reported herein is reported up to 116 days after boost vaccination. As a comparative cohort, we performed a retrospective analysis of sera from individuals who received homologous BNT162b2 vaccination with a 3-week interval (14 individuals aged 25-65, median 42; Appendix Table 1).^24^

### Ethics

The study was approved by the ethics committee of Ulm university (99/21) and performed in compliance with the approval. Previously described sera from homologous BNT162b2 vaccinated individuals,^24^ taken under approval by the ethics committee of Ulm university (31/21), were re-analysed for this study. All participants gave written informed consent.

### Collection of serum and PBMC samples

Hospital employees who were scheduled for vaccinations volunteered for regular blood donations. Participants received a heterologous vaccination regimen because after their ChAdOx1 nCoV-19 prime vaccination, the German Standing Committee on Vaccination (STIKO) had changed the recommendation for individuals < 60 years of age to receive an mRNA vaccine as boost vaccination to avoid risk of thrombotic complications.^13^^,^^16^ At days -2/0 before vaccination, days 15-16, 30-37, 53-57 after ChAdOx1 nCoV-19 vaccination, and days 6-11, 14-19, 38-48 and 93-116 after heterologous BNT162b2 boost, blood was drawn into S-Monovette® Serum Gel (Sarstedt) or S-Monovette® K3 EDTA tubes. Sera from individuals vaccinated twice with BNT162b2 were obtained 13-15 days after the second dose (Appendix Table 1); these sera were previously described and re-analysed for this study.^24^ Serum gel collection tubes were centrifuged at 1,500 × g at 20°C for 15 min, aliquoted and stored at -20°C until further use. Peripheral blood mononuclear cells (PBMCs) were obtained from EDTA tubes using density gradient centrifugation by Pancoll human (Pan Biotech, Germany), and erythrocytes removed by ACK lysis buffer (Lonza, Walkersville, MD, U.S.A). Mononuclear cells were counted for viability using a Countess II Automated Cell Counter (Thermo Fisher) with trypan blue stain and were cryopreserved in aliquots of up to 1 × 10^7^ cells in 10% DMSO in heat-inactivated FCS.

### Vaccine reactogenicity

Solicited adverse reactions (SAR) were self-reported by the participants via questionnaire following prime and boost vaccination. Participants were asked to list symptoms, their duration (< 1 h, few hours, one day or more than one day) and severity (mild (grade 1), moderate (grade 2), severe (grade 3)). Grading criteria were adapted from the US Department of Health and Human Services CTCEA (Common Terminology Criteria for Adverse Events, v4.03),^25^ with grade 1-2 being considered for some symptoms, grade 1-3 for most. For calculation of cumulative SAR (cSAR) scores, the grades of all symptoms listed were summed up, with an additional score point added for each symptom that was experienced for more than one day (0-4).

### Determination of antibody titres

IgG and IgA levels in serum were determined by anti-SARS-CoV-2 assay (Euroimmun), an ELISA which detects antibodies against the SARS-CoV-2 S1 spike domain. The assay was performed according to the manufacturer's instructions. Briefly, serum samples were diluted 1:10 in sample buffer and pipetted into rSARS-CoV-2 spike precoated strips of eight single wells of a 96-well microtitre plate. After incubation for 60 min at 37°C, wells were washed three times, peroxidase-labelled anti-IgG or anti-IgA added and incubated. After 30 min, three additional washing steps were performed before substrate was added and incubated for 15-30 min in the dark. Thereafter, stop solution was added, and optical density (OD) values measured on a POLARstar Omega plate reader (BMG LABTECH, Ortenberg, Germany) at 450 nm corrected for 620 nm. Finally, OD ratios were calculated based on the sample and calibrator OD values, where a ratio <0.8 was considered to be negative and >1.1 to be positive. To quantify antibody responses, IgG and IgM were measured as units per ml (U/ml) that correlates with the WHO standard unit for the SARS-CoV-2 binding antibody units per ml (BAU/ml). To this end, serum was analysed using the commercial electrochemiluminescence Elecsys Anti-SARS-CoV-2 S immunoassay (Roche, Mannheim, Germany) by a cobas® e801 immunoassay analyser according to the manufacturer's instructions (Roche).

### Surrogate SARS-CoV-2 neutralization test

Prevention of SARS-CoV-2 spike RBD interaction with ACE2 by sera was evaluated by SARS-CoV-2 Surrogate Virus Neutralization Test Kit (GenScript) according to the manufacturer's instructions. To this end, sera were incubated with a peroxidase-conjugated RBD fragment and the mixture added to a human ACE2 coated plate, and unbound RBD washed away. Thereafter, substrate was added and the reaction stopped by stopping reagent. ODs at 450 nm were measured at a microplate reader. The inhibition score compared to the negative control was calculated as percentages. Scores <20% were considered negative and scores >20% positive.

### Cell culture

Vero E6 (African green monkey, female, kidney*;* CRL-1586, ATCC, RRID:CVCL_0574) cells were grown in Dulbecco's modified Eagle's medium (DMEM, Gibco) which was supplemented with 2.5% heat-inactivated fetal calf serum (FCS), 100 units/ml penicillin, 100 µg/ml streptomycin, 2 mM L-glutamine, 1 mM sodium pyruvate, and 1x non-essential amino acids. HEK293T (human, female, kidney; ACC-635, DSMZ, RRID: CVCL_0063) cells were grown in DMEM with supplementation of 10% FCS, 100 units/ml penicillin, 100 µg/ml streptomycin, 2 mM L-glutamine. All cells were grown at 37°C in a 5% CO_2_ humidified incubator. Cell lines were recently purchased from the indicated companies and used without further authentication. All cell lines were regularly tested for mycoplasma contamination and remained negative.

### Preparation of pseudotyped particles

Expression plasmids for vesicular stomatitis virus (VSV, serotype Indiana) glycoprotein (VSV-G) and SARS-CoV-2 spike variants B.1.1.7, B.1.351, B.1.617.1, and B.1.617.2 (codon-optimized; with a C-terminal truncation for increased pseudovirus packaging) have been described elsewhere.^24^^,^^26^^,^^27^ Transfection of cells was carried out by Transit LT-1 (Mirus). Rhabdoviral pseudotype particles were prepared as previously described.^28^ A replication-deficient VSV vector in which the genetic information for VSV-G was replaced by genes encoding two reporter proteins, enhanced green fluorescent protein and firefly luciferase (FLuc), VSV∗ΔG-FLuc^29^ (kindly provided by Gert Zimmer, Institute of Virology and Immunology, Mittelhäusern, Switzerland) was used for pseudotyping. One day after transfection of HEK293T cells to express the viral glycoprotein, they were inoculated with VSV∗ΔG-FLuc and incubated for 1-2 h at 37°C. Then the inoculum was removed, cells were washed with PBS and fresh medium added. After 16-18 h, the supernatant was collected and centrifuged (2,000 × g, 10 min, room temperature) to clear cellular debris. Cell culture medium containing anti-VSV-G antibody (I1-hybridoma cells; ATCC no. CRL-2700) was then added to block residual VSV-G-containing particles. Samples were then aliquoted and stored at -80°C.

### Pseudovirus neutralisation assay

For pseudovirus neutralisation experiments, Vero E6 were seeded in 96-well plates one day prior (6,000 cells/well). Heat-inactivated (56°C, 30 min) sera were serially titrated (4-fold titration series with 7 steps + buffer only control) in PBS, pseudovirus stocks added (1:1, v/v) and the mixtures incubated for 30 min at 37°C before being added to cells in triplicates (final on-cell dilution of sera: 20, 80, 320, 1,280, 5,120, 20,480, 81,920-fold). After an incubation period of 16-18 h, transduction efficiency was analysed. For this, the supernatant was removed, and cells were lysed by incubation with Cell Culture Lysis Reagent (Promega) at room temperature. Lysates were then transferred into white 96-well plates and luciferase activity was measured using a commercially available substrate (Luciferase Assay System, Promega) and a plate luminometer (Orion II Microplate Luminometer, Berthold). For analysis of raw values (RLU/s), background signal of an uninfected plate was subtracted and values normalized to pseudovirus treated with PBS only. Results are given as serum dilution resulting in 50% virus neutralization (NT50) on cells, calculated by nonlinear regression ([Inhibitor] vs. normalized response – Variable slope) in GraphPad Prism Version 9.1.1.

### Determination of CD4^+^ and CD8^+^ SARS-CoV-2 spike-specific T cell responses by intracellular cytokine staining (ICS)

Cryopreserved PBMCs of study participants were thawed and rested overnight at 37°C with 1 µl/ml of DNAse (DNase I recombinant, RNase-free (10,000 U) Roche), in RPMI medium supplemented to contain a final concentration of 10% FCS (Corning Life Sciences/Media Tech Inc, Manassas, VA), 10 mM HEPES, 1x MEM nonessential amino acids (Corning Life Sciences/Media Tech Inc, Manassas, VA), 1 mM sodium pyruvate (Lonza, Walkersville, MD, U.S.A), 1 mM penicillin/streptomycin (Pan Biotech, Germany) and 1 × 2-mercaptoethanol (GIBCO, Invitrogen, Carlsbad, CA, U.S.A). Stimulation of PBMCs for detection of cytokine production by T cells was adapted from Kasturi *et al.*, 2020.^30^ Briefly, 1 × 10^6^ PBMCs were cultured in 200 μl final volume in 96-well U bottom plate in the presence of anti-CD28 (1 μg/ml) and anti-CD49d (1 μg/ml) (Biolegend) under the following conditions: a) negative control with DMSO, b) SARS-CoV-2 spike peptide pools (1-315 peptides from Wuhan-Hu-1, Alpha (B.1.1.7), Beta (B.1.351), Gamma (P.1), and Delta (B.1.617.2) SARS-CoV-2 spike, JPT Germany) at a final concentration of 2 μg/ml, c) PMA/ionomycin, d) CEFX Ultra Super Stim peptide pool (176 peptide epitopes for a broad range of HLA subtypes and different infectious agents, JPT Germany) at a final concentration of 2 μg/ml. Cells were cultured for 2 hours before adding Brefeldin A at 10 μg/ml (Sigma-Aldrich, St Louis, MO) for an additional 5 hours. Cells were then washed with PBS, and stained for dead cells (Live/ Dead Fixable; Aqua from Thermo Fisher) in PBS at room temperature for 10 minutes. Without washing, cells were incubated with surface antibody cocktail (prepared in 1:1 of FACS buffer and brilliant staining buffer) for 30 minutes at room temperature with BV510-anti-human CD14 (clone M5E2), BV510-anti-human CD19 (clone HIB19), AF700 anti-human CD3 (clone OKT3), BV605 CD4 (clone OKT4), PerCP-Cy5.5 CD8 (clone RPA-T8) from Biolegend. Next, cells were fixed using Cytofix/Cytoperm buffer (BD Biosciences, CA) for 20 minutes at room temperature, and then kept in FACS buffer at 4°C overnight. 1x Perm/Wash (BD Biosciences, CA) was used for cells permeabilization for 10 minutes at room temperature and followed by intracellular staining for 30 minutes at room temperature with AF647 anti-human IFNγ (clone 4S.B3) and AF488 anti-human IL-2 (clone MQ1-17H12) from Biolegend, and PE/Cy7 anti-human TNFα (clone Mab11) from Thermo Fisher Scientific. Up to 100,000 live CD3^+^ T cells were acquired on a LSRFortessa flow cytometer (BD Biosciences), equipped with FACS Diva software. Analysis of the acquired data was performed using FlowJo software (version 10.7.1). The background from each participant was removed by subtracting the % of spike^+^ cells to the % of DMSO^+^ cells.

### Statistics

The SARS-CoV-2 convalescent individual was excluded in all statistical analyses. Data of participants that dropped out were excluded from evaluation. Non-parametric Spearman rank correlation was used to check for possible associations at single blood sample measurements. A paired t-test was used to compare the adverse event scores calculated for each participant after both vaccinations. For this, the individual mean differences were checked for normal distribution by means of QQ-plots and histograms. A comparison of participants receiving heterologous vaccination with controls who received homologous BNT162b2 vaccinations after the last blood sample measurements was done by the Mann-Whitney-U test because of skewed distributions for neutralization scores as well as IgM/IgG measurements. Longitudinal antibody and T cell measurements were analysed by means of a mixed linear regression model including a random intercept in order to account for the repeated measures structure of the underlying data. The mixed linear model approach enabled to simultaneously account for possible confounding due to participants' sex and for the presence of missing data.^31^ Therefore, no formal imputation of missing interim values was required. A two-sided alpha error of 5% was applied to analyses. The repetitive measurement and analysis of longitudinal samples provided by a cohort of 26 participants allows robust statistical evaluation. All analyses were done by GraphPad Prism version 9.1.1 for Windows, GraphPad Software, San Diego, California USA, www.graphpad.com, R (version 4.0.1) and SAS (version 9.4).

### Role of funding source

Funders had no role in study design, data collection, data analyses, interpretation, or writing of the report.

## Results

Prior to vaccination, one participant of our cohort of 26 individuals had a history of SARS-CoV-2 infection (Table 1).

Reactogenicity following prime and boost vaccination was evaluated in all study participants by examining self-reported solicited local and systemic symptoms according to a standardized questionnaire. Symptom severity (mild, moderate, severe) and duration (<1 hour, few hours, ∼1 day, > 1 day) is reported for each individual participant (Figure S1a) and percentage of participants (Figurere 1a,b).

**Figure 1:**
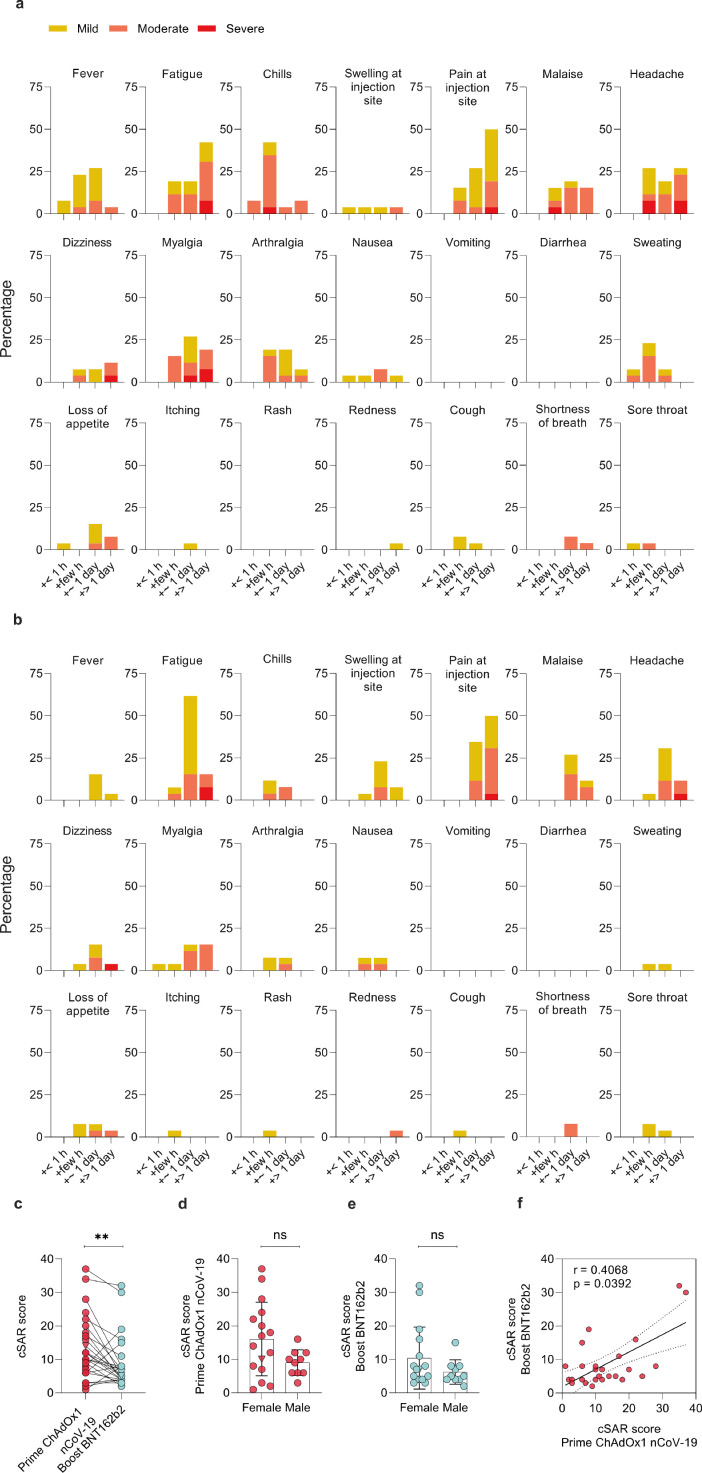
Solicited adverse reactions following ChAdOx1 nCoV-19 prime and BNT162b2 boost vaccination. Percentages of n=26 participants with individual symptoms following prime (a) or boost (b) vaccination. Severity is graded on a scale of 1-2 (for some symptoms) or 1-3 (for most), as adapted from the Common Terminology Criteria for Adverse Events (US Department of Health and Human Services, Version 4.03).^25^ (c) Cumulative solicited adverse reaction (cSAR) scores of all participants following prime and boost vaccination. For calculation of cSAR scores, symptom gradings are summed and an additional score point is added for symptoms lasting more than 24 h. Paired two-tailed t-test compares prime and boost vaccination. (d,e) Analysis of cSAR scores by participant sex (Mann-Whitney-U test). (f) Spearman correlation of cSAR scores following prime and boost vaccination. The SARS-CoV-2 convalescent individual (triangle) was excluded in all statistical analyses.; ns not significant; ** p < 0.01

Both, prime and boost vaccination, induced mild to moderate solicited adverse reactions in most participants with 88.4% (23/26) reporting at least one mild or moderate symptom following the prime; 23/26 (88.4%) and 21/26 (80.8%) reporting at least one mild or moderate symptom following the mRNA boost (Figurere 1a,b). Most common symptoms after prime vaccination with ChAdOx1 nCoV-19 were pain at the injection site (92.3%), fatigue (80.8%), headache (73.1%), chills (61.5%), myalgia (61.5%) and fever (61.5%). Following boost vaccination with BNT162b2, most participants again reported pain at the injection site (84.6%) and fatigue (84.6%), but chills (19.2%), myalgia (38.5%) and fever (19.2%) were less common. 23% of participants (6/26) reported at least one severe symptom following prime, 15.4% (4/26) after boost. Fatigue (7.7%) and headache (15.4% for prime, 3.8% for boost) were amongst symptoms reported as more severe for both doses, while myalgia was reported by 11.5% of participants following prime, but none after boost. Of note, after ChAdOx1 nCoV-19 prime, no participant developed platelet factor 4 autoantibodies^32^ (Table 1).

To get an estimate about how the overall reactogenicity was perceived, we compared cumulative solicited adverse reaction (cSAR) scores, and found that reactogenicity following prime with ChAdOx1 nCoV-19 was significantly (p = 0.008; paired t-test) higher than following boost with BNT162b2 (cSAR score median 11 and 6 respectively, Figurere 1c). Individually, most participants (19/26, 73.07%) had milder reactions to boost compared to prime, similar as described for a homologous ChAdOx1 nCoV-19 vaccination.^6^ 6/26 (23.07%) of participants described more severe reactions to boost vaccination (Figure S1b). In this cohort of 26 participants, a non-significant trend (p > 0.1; Mann-Whitney-U test) towards higher cSAR scores reported by female participants was seen for both boost and prime vaccinations (Figurere 1d,e). No correlation was observed between reactogenicity and age (Figure S1c,d; |r| < 0.3; Spearman). Individual reactogenicity towards prime and boost vaccination showed a modest but significant correlation (Figurere 1f, r = 0.41; p = 0.039; Spearman).

We collected sera from participants 2 days before (-2) or on the day of (0) vaccination, and at days 15–16, 30–37, and 53–57 after ChAdOx1 nCoV-19 prime, and days 6–11 and 14–19 after BNT162b2 boost (64–65 or 72–73 after prime, respectively) to determine antibody responses (Figurere 2). Already 15–16 days after prime, 19/25 (76%) participants showed detectable anti-SARS-CoV-2-spike-IgG levels and 17/25 (65%) detectable IgA levels (Figurere 2a,b). IgG levels peaked after 30–37 days and were detectable in 24/25 (96%) participants. Until days 53–57, IgG levels slightly decreased, consistent with previous results after single ChAdOx1 nCoV-19 dose.^5^^,^^6^ IgA values were highest at days 15–16 and became undetectable in 24 (92%) participants at days 53–57. Notably, only 6–11 days after the BNT162b2 boost, IgG was detectable in all (100%) and IgA in 23/25 (92%) participants. Until day 14–19 after boost (72–73 post ChAdOx1 nCoV-19), IgG and IgA were detectable in all participants. This corresponds to a 3.7-fold increase in median IgG levels from pre-boost to 2 weeks post-boost (p < 0.0001; mixed linear regression model). We next quantified cumulative anti-SARS-CoV-2-spike-IgM and IgG concentrations and detected median antibody levels of 3.39 (range 0-2,126) units per ml (U/ml) 15–16 days after prime vaccination in 22/25 (88%) participants (Figurere 2c). From days 30–37 on, IgM and IgG were detected in all participants and medians continuously increased to 28 (1.86-1,436) and 63.9 (4.27-1,005) U/ml after days 30–37 or 53-57, respectively. After BNT162b2 boost, titres increased 134-fold to 8,614 (126 – 24,831; p < 0.0001; mixed linear regression model) at days 6–11 and 135-fold to 8,815 (1,206 – 19,046; p < 0.0001; mixed linear regression model) 14–19 days after the second dose. Notably, the resulting titres were 8.1-fold higher than those determined for sera obtained after 13–15 days of a homologous BNT162b2 boost (p < 0.0001; Mann-Whitney-U test) (individuals with median age 41 (25-55; Appendix Table 1) and median titres 1,086; range 498-3,660). Cumulative IgM/G titres correlated with IgG titres at each timepoint analysed post prime (Figure S2a, Appendix Table 2). In addition, we followed up antibody titres over the course of 13-17 weeks (93-116 days) after boost, and confirmed that they are decreasing. However, they maintained a median titre of 2,039 (235 - 5,926) that was still 1.9-fold higher than in homologous BNT162b2 vaccinated individuals 2 weeks post boost (p = 0.038; Mann-Whitney-U test).

**Figure 2:**
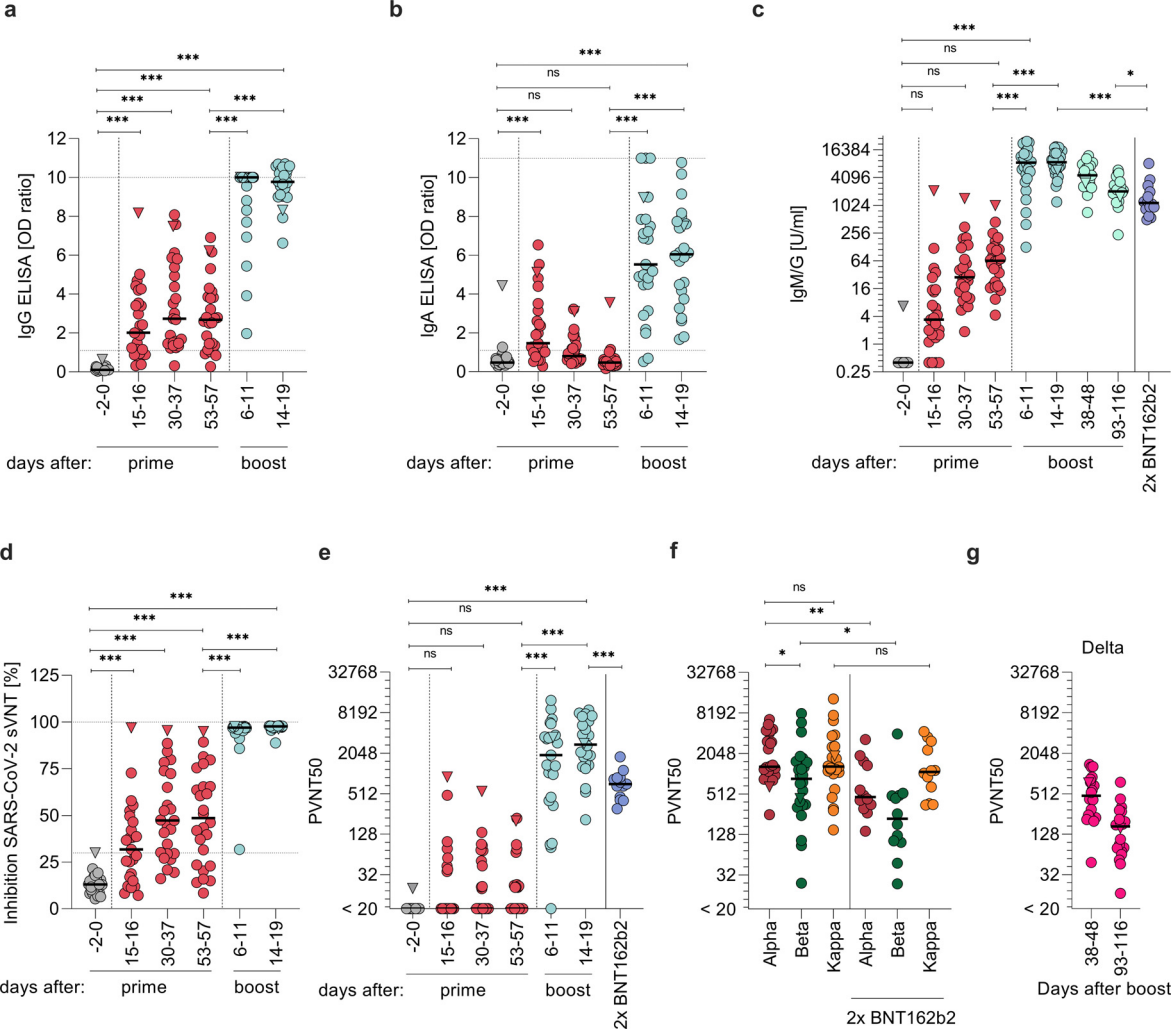
Humoral response. Quantification of anti-SARS-CoV-2 S1 spike domain (a) IgG and (b) IgA titres. (c) Quantification of anti-SARS-CoV-2 spike IgG and IgM responses as units per ml (U/ml) by immunoassay. (d) SARS-CoV-2 surrogate virus ACE2 neutralization test. (e) VSV-based Alpha SARS-CoV-2 spike pseudovirus neutralization assay. (f) VSV-based Alpha, Beta, and Kappa SARS-CoV-2 spike pseudovirus neutralization assay from sera obtained 14-19 days post boost. (g) VSV-based Delta SARS-CoV-2 spike pseudovirus neutralization assay from sera obtained 38-48 and 93-116 days post boost. Titres expressed as serum dilution resulting in 50% pseudovirus neutralization (PVNT50) were obtained from triplicate infections. Triangle indicates SARS-CoV-2 convalescent individual, who was excluded from all statistical analyses. Grey symbols indicate datapoints pre-vaccination, red datapoints indicate datapoints after prime and light-blue after boost vaccination. Dark-blue indicates samples of participants with homologous BNT162b2 prime-boost regimen. Dashed horizontal lines indicate upper and lower limit of detection/cut-off, respectively. Dashed vertical lines indicate prime and boost vaccination. Samples were obtained from n=26 participants. Longitudinal antibody measurements were analysed by means of a mixed linear regression model. Mann-Whitney-U test compares ChAdOx1 nCoV-19 and BNT162b2 titres and variants *** p < 0.0001, ** p < 0.01, * p < 0.05, ns = not significant

Sera were also evaluated for their potential to inhibit SARS-CoV-2-spike-receptor binding domain/ACE2 interaction using a surrogate virus neutralization test (sVNT) (Figurere 2d). 15–16 days after ChAdOx1 nCoV-19 administration 13/25 (52%) participant sera showed ACE2 neutralizing activity, correlating significantly with IgG and IgM/G titres (Figure S2a, Appendix Table 2). Median neutralization activity of the positive sera was 46% (range 32-97%). Until days 53–57, the number of participants with neutralizing sera increased to 19/26 (73%) and the median ACE2 neutralization to 62% (range 32-95%), again in correlation with IgG and IgM/G values (Figure S2a, Appendix Table 2). After BNT162b2 boost, all participants showed potent neutralization with a median of 97% (range 32-98%) after 6–11 days, and 98% (range 89-98%) after 14–19 days suggesting a strong and functional antibody response after heterologous vaccination in all participants.

The potency of neutralizing activity was further quantified using vesicular stomatitis virus (VSV)-based pseudoviruses carrying the SARS-CoV-2 spike protein of the SARS-CoV-2 Alpha (B.1.1.7) variant. This system faithfully recapitulates SARS-CoV-2 entry into cells and its inhibition.^26^^,^^33^^,^^34^ 15–16 days after ChAdOx1 nCoV-19 prime, neutralizing titres ranging from 36-906 were detectable in 8/25 (32%) participants (Figurere 2e). The number of participants with detectable neutralization activity increased to 12/25 (48%) individuals at days 30–37 with a median neutralization titre of 74 (range 20-552) in responders, which slightly decreased until days 53–58. Two weeks after the BNT162b2 boost, neutralizing activities were detected in all participants with a median titre of 2,744 (range 209-8,985). Of note, while for some individuals the titres further increased from week 1 to week 2 after BNT162b2 boost, it plateaued at titres > 1,000 for others (Figure S3). At all time-points, neutralizing activity correlated with IgG or IgM/G titres (Figure S2a, Appendix Table 2). Remarkably, the median titre of these individuals was 3.9-fold higher than the median titre two weeks post boost of 14 individuals vaccinated with BNT162b2 in a homologous regimen^24^ (709; range 305-1,806; p < 0.0001; Mann-Whitney-U test) suggesting stronger humoral protection after the heterologous vaccination. Multivariable analyses did not indicate sex or age as an important confounder for this effect (p > 0.05). Of note, a SARS-CoV-2 convalescent individual (triangle symbol) showed a strong response after the first dose in all assays, high IgG, IgA or IgM/G values, most effective ACE2-neutralization and a high neutralization titre of 906 15 days after prime that decreased to 201 at day 53 (Figurere 2a-e).

Additionally, we evaluated the neutralizing activities of sera obtained 2 weeks post full vaccination against the immune evading variants Beta (B.1.351) and Kappa (B.1.617.1). Pseudovirus entry driven by Beta spike was neutralized with 2-fold lower potency (p < 0.021; Mann-Whitney-U test) compared to Alpha spike. However, it was still entirely blocked at higher doses with a median titre of 1,297 (range 252 - 6,523). Neutralization of the Kappa spike was not reduced compared to Alpha spike (median titre of 1,309; range 150 – 13,252) (Figurere 2f; p = 0.65; Mann-Whitney-U test). Sera of individuals receiving two BNT162b2 immunizations showed lower neutralizing titres against all spike variants tested (Figurere 2f), suggesting stronger humoral protection after the heterologous vaccination also against VOCs. To analyse the neutralizing capacity of the sera against the dominant and thus most relevant Delta variant, we collected follow-up sera of the participants in week 5-7 and 13-17 (days 38-48 and 93-116) after the boost. We found that all sera neutralized the Delta variant, however, with lower titres than previously determined against Alpha and decreasing efficiency from 5-7 to 13-17 weeks post boost (Figurere 2g). Again, these titres correlated well with the IgM/IgG concentrations (Figurere 2c; Figure S2b). In both cohorts , no correlation of neutralizing antibody titre (PVNT50) with age nor sex were observed (Figure S4; |r| < 0.3, Spearman; p > 0.1, Mann-Whitney-U test).

To evaluate cellular immunity, we isolated peripheral blood mononuclear cells from blood samples provided by 21/26 participants before ChAdOx1 nCoV-19 prime, and 6–11 and 14-19 days post BNT162b2 boost (64-65 and 72-73 days post prime), considered as full vaccination according to the vaccination schedule. Cells were exposed to SARS-CoV-2 spike-spanning peptide-pools and analysed for intracellular cytokines TNFα, IFNγ, and IL-2 to determine spike-specific CD4^+^ and CD8^+^ T cell responses (Figurere 3, S5, S6). 6-11 days after boost, 68% of participants showed IFNy, TNFα, and/or IL-2 CD4^+^ T cell responses upon stimulation with Wuhan-Hu-1 SARS-CoV-2 spike peptides (Figurere 3a). After full vaccination, CD4^+^ T cells producing IFNγ (median 0.055%, range 0.018-0.168), IL-2 (median 0.055%, range 0-0.134) or TNFα (median 0.057%; range 0.01 – 0.193) were significantly increased upon stimulation as compared to the pre-vaccination baseline (p < 0.05; mixed linear regression model). Spike-reactive CD4^+^ T cells were detected in 95% suggesting that most individuals developed a robust spike-specific T helper 1 (TH1) CD4^+^ T cell response post BNT162b2 boost. In addition, we observed an increase of spike-specific CD8^+^ T cells predominantly producing IFNγ (median 0.092%, range 0 - 0.665) and TNFα (median 0.055%, range 0 – 0.375) compared to pre-vaccination baseline samples in all previously uninfected participants (Figurere 3a; p < 0.001; mixed linear regression model). Levels of spike-specific CD8^+^ T cells producing IL-2 (median 0.01%, range 0-0.052) were lower, which is in agreement with responses reported after homologous BNT162b2 vaccination described in a preprint.^8^ We then compared the response to SARS-CoV-2 spike variants Alpha, Beta, and Gamma (P.1) at day 6-11 after boost, and found that both CD4^+^ and CD8^+^ T cells showed IFNγ responses to all spike variants (Figurere 3b), however less pronounced for TNFα and IL-2 (Figure S6 c,d). Upon emergence and rapid spread of SARS-CoV-2 Delta, we analysed the reactivity of T cells obtained day 14-19 post boost against this VOC. 94% of participants showed increased responses of CD4^+^ T cells upon stimulation with Delta spike peptides and all participants had Delta spike specific CD8^+^ T cells (Figurere 3c). Thus, T cell reactivity against all five spike variants was found in all participants. Of note, reactivity of the CD4^+^ and CD8^+^ T cells of the convalescent individual did not increase after vaccination (Figurere 3a), indicating pre-existing cellular immunity. In conclusion, these findings show a robust humoral and cellular immune response after heterologous vaccination.

**Figure 3:**
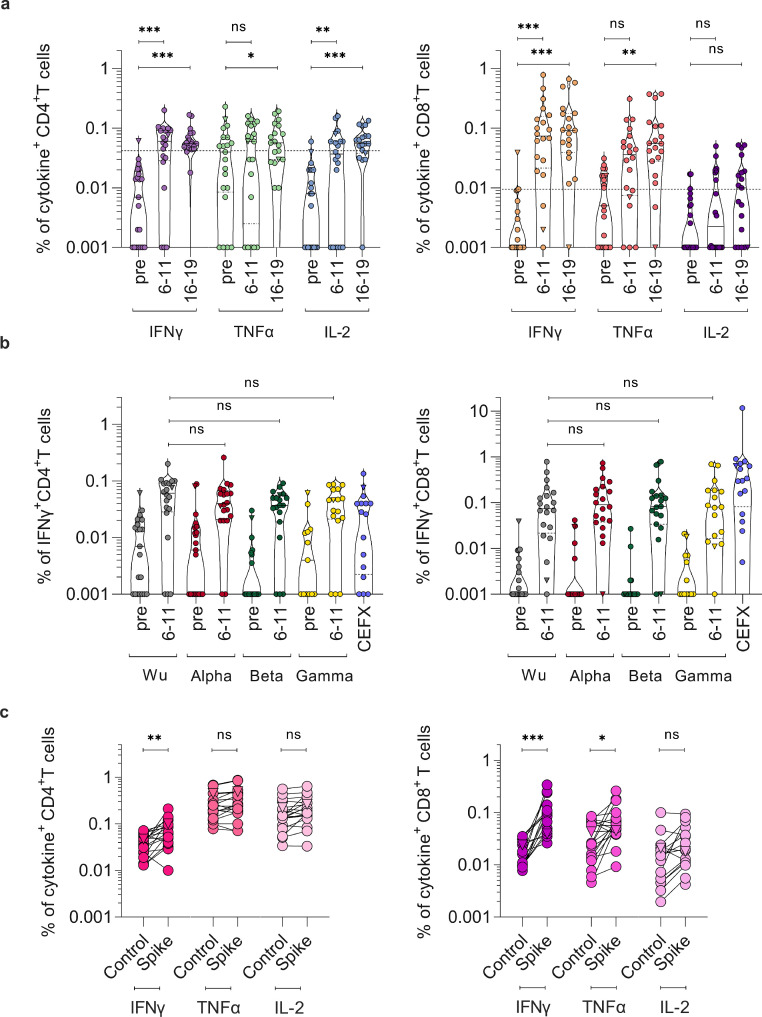
SARS-CoV-2 spike-specific CD4^+^ and CD8^+^ T cell responses. PBMCs from day -2/0 pre-vaccination (pre) and day 6-11 or 16-19 post boost (64-65 or 72-73 post prime) of n = 21 study participants were stimulated with (a) SARS-CoV-2 Wuhan-Hu-1 (Wu) spike peptide-pool and cytokine production determined by flow cytometry. Cytokine^+^ T cells were background-corrected for unstimulated cells. Values lower than median plus one standard deviation of pre-vaccination (0.04% for CD4, 0.01% for CD8) were considered negative. (b) PBMCs from day 0-2 pre and day 6-11 post boost (day 64-65 post prime) were stimulated with SARS-CoV-2 spike peptide pools derived from Alpha, Beta, or Gamma, or of epitopes of different infectious agents (CEFX) and compared with Wuhan-Hu-1 from (a). (c) PBMCs from day 14-19 post boost (72-73 post prime) were stimulated with a SARS-CoV-2 Delta spike peptide-pool or control (DMSO) (control) and cytokine production determined by flow cytometry. Triangle symbol indicates SARS-CoV-2 convalescent individual, where cytokine release was already high in absence of stimulation. Longitudinal T cell responses were analysed by means of a mixed linear regression model. Mann-Whitney-U test compares cytokine-positive cells at 6-11 days post boost (64-65 days after prime) upon stimulation with different SARS-CoV-2 spike variants. Unpaired t-test compares T cell responses upon control versus Delta spike-peptide treatment. *** p < 0.0001, ** p < 0.01, * p < 0.05, ns = not significant.

## Discussion

Heterologous prime-boost regimens have already been performed in several countries even though clinical studies had not been available at the time and studies on effectiveness have only recently begun to be evaluated.^35^^,^^36^ Based on the regulatory approvals for ChAdOx1 nCoV-19 and mRNA vaccines, the interval between prime and boost vaccinations ranges between 4-12 weeks.^37^, ^38^, ^39^ For ChAdOx1 nCoV-19, a 12 week interval has been shown to result in stronger immune responses,^40^ likely because the immunity against the vector wanes and the immunological memory matures. Accordingly, in Germany heterologous vaccinations were typically performed after 12 weeks. Existing vector immunity, however, is irrelevant in the context of a mRNA boost vaccination, on which basis our cohort received the boost after 8 weeks. This heterologous ChAdOx1 nCoV-19/BNT162b2 vaccination elicited strong IgM/G and IgA responses, potent neutralizing antibodies and T cell responses in all previously uninfected participants, while solicited adverse reactions to vaccination were as expected for a prime ChAdOx1 nCoV-19 vaccination and reduced following heterologous BNT162b2 boost.

In a homologous ChAdOx1 nCoV-19 vaccination regimen with an 28 day interval, the boost dose is associated with lower reactogenicity than the prime dose.^6^ A previous study showed that a heterologous ChAdOx1 nCoV-19 prime BNT162b2 boost vaccination schedule with 4-week interval results in stronger reactogenicity after boost,^20^ whereas studies with an 8- to 12-week interval did not confirm this effect.^17^, ^18^, ^19^ We did not directly compare different vaccination schemes, as our comparative homologous BNT162b2 vaccination cohort was analysed retrospectively for serology and not polled for reactogenicity. Thus, we cannot draw definitive conclusions on quantitative differences, which might also depend on cohort age.^6^ With an 8-week interval, we observed an overall milder reactogenicity following heterologous boost with BNT162b2 than after initial prime vaccination with ChAdOx1 nCoV-19 and no serious adverse events.

Our immunological data suggest that a heterologous vector-based/mRNA prime-boost schedule would be highly effective in preventing COVID-19, as neutralizing antibody levels correlate with immune protection from symptomatic SARS-CoV-2 infection^41^ and CD8^+^ T cell responses have been associated with a mild disease course.^42^^,^^43^ Endpoint neutralizing antibody titres determined 2 weeks post boost were ∼4-fold higher than those detected upon homologous BNT162b2 vaccinations suggesting stronger humoral protection (Figurere 2e). Even though our statistical analyses do not identify possible confounders, this outcome might also depend on sex, age or the vaccination intervals between the two groups as the homologous BNT162b2 cohort was ∼10 years older in age, had higher fraction of male participants and was vaccinated in a three-week interval (Table 1; Appendix Table 1). Yet, this observation is in agreement with other studies^19^^,^^21^^,^^44^ and suggests that ChAdOx1 nCoV-19 / BNT162b2 prime-boost vaccination elicits particularly potent immune responses.^17^^,^^18^^,^^45^ As reported for homologous vaccinations,^46^ titres also decrease over time after heterologous vaccination. However, they remained high for three months post boost suggesting robust durable protection. Notably, the results might also be influenced by the young cohort age. Circumvention of vector immunity might also contribute to this high immunogenicity.^35^ The BNT162b2 encoded spike sequence contains a two-proline mutation not present in ChAdOx1 nCoV-19, which stabilizes the spike protein in a pre-fusion confirmation.^10^^,^^47^ It is tempting to speculate that altered spike conformations between the vaccinations might improve humoral responses.

Neutralizing activity towards VOC Beta, previously reported to show partial evasion of vaccination-induced antibodies,^24^^,^^48^^,^^49^ was slightly decreased compared to Alpha following heterologous vaccination, which agrees with recent data.^21^ However, the titre was still higher than for Alpha after two doses of BNT162b2. Neutralization of emerging Kappa, reported to be the most efficient neutralization-evading strain of the B.1.617 lineage,^44^ was not reduced compared to Alpha. Previous studies addressing heterologous vaccinations indicated that the Delta variant is equally sensitive to neutralization as Alpha.^22^^,^^23^^,^^45^ We observed that in correlation with the IgM/IgG titres, neutralizing titres against Delta are decreasing over the course of 13-17 weeks but remain detectable in all analysed sera at the latest time points. This confirms a good prediction by IgM/IgG titres and indicates durable protection from the most prevalent Delta variant. Breakthrough infections of vaccinated individuals are associated with low neutralizing titres,^50^ and a decline of the titre over time results in less effective SARS-CoV-2 neutralization.^46^^,^^51^ Thus, the fact that neutralizing titres after heterologous boost are high suggests efficient initial protection from breakthrough infections, also by the Delta variant. Ongoing and future studies addressing the decay kinetics will allow to determine the time after which a second boost might be most beneficial.

Secretory IgA responses at the mucosal site of SARS-CoV-2 entry are of particular interest with regard to prevention of virus transmission and (re-)infection.^52^ We detected a general increase in serum IgA levels with strong variation between participants and a decrease over time after prime vaccination. Future studies, especially assessing IgA and secretory IgA levels and persistence at mucosal entry sites after boost are warranted to analyse potential mucosal protection after vaccination.

In all participants SARS-CoV-2 specific CD8^+^ or CD4^+^ T cells were detected 2 weeks after full vaccination. These effects were similar to those reported after a single ChAdOx1 nCoV-19 dose^5^ and after homologous BNT162b2 vaccination as reported in a preprint.^8^ This suggests that T cell responses are similarly effective after heterologous vaccination. Importantly, all donors showed some degree of T cell reactivity against the prevalent Delta variant. Similarly, responses against Alpha and most humoral immunity-evading VOCs Beta and Gamma were detectable suggesting a broad immune response. This is in line with previous findings that variants escaping humoral immunity are still recognized by reactive T cells indicating broad protection from SARS-CoV-2 on a cellular level,^10^^,^^53^ also after heterologous vaccination.

In line with previous reports,^54^^,^^55^ a single prime vaccination dose already elicited strong antibody responses in an individual participant who was previously tested SARS-CoV-2 positive. In this case, the observed neutralizing titres 2 weeks after prime were as high as the median titre of those receiving the homologous BNT162b2 vaccination. However, titres (IgM/G) further increased 8-fold after boost, suggesting that prime-boost might provide more potent and longer lasting protection also in convalescent patients. This agrees with a recent study^56^ and seems important considering that the currently dominating Delta variant is causing breakthrough infections.^50^

In conclusion, a heterologous vaccination regimen of ChAdOx1 nCov-19 prime, followed by BNT162b2 boost after 8 weeks for participants with a median age of 30.5 years was tolerable and effective. These results are encouraging and of significant interest for policy makers, physicians as well as individuals who have received or will receive this combination. This regimen provides flexibility for future vaccination strategies and will be useful for vaccine schedules during shortages. The perspective that heterologous vaccination induces neutralizing titres predictive of protection should be considered as a potential strategy to elicit particularly strong immune responses, e.g. in immunocompromised, highly exposed individuals, against Delta and upcoming VOCs, and for third doses of vaccination after previous homologous prime-boost regimen or vaccine updates against COVID-19. It will be interesting to clarify whether different spike conformations or of different variants are particularly effective. Similarly, whether other vector- or mRNA-based vaccine combinations or those based on other technologies are as effective needs to be addressed in future studies.

### Limitations

The study cohort of 26 participants is not large, but due to longitudinal sampling a comprehensive statistical evaluation could be performed. Self-reported adverse reactions can be subject to recall bias and indicated grades of severity are subject to personal and individual sensation. With a median age of 30.5 (range 25 - 46) years, the results do not provide information on the elderly. However, our study offers insight into how the younger age group reacts to a heterologous vaccination regimen. This is of high significance, because individuals younger than 60 have regularly been primed with ChAdOx1 nCov-19 and are now offered heterologous boost vaccination. Our study group received the second vaccination after 8 weeks, which is within the range of recommendation of 4-12 weeks for homologous ChAdOx1 nCov-19 vaccination. Humoral responses determined 15-17 after boost suggest ongoing protection but do not allow conclusions about longer-lasting immunity.

## Declaration of interest

The authors have nothing to disclose.

## Supplementary materials

Image, application 1

Image, application 2
